# Microencapsulation and Nanoencapsulation Using Supercritical Fluid (SCF) Techniques

**DOI:** 10.3390/pharmaceutics11010021

**Published:** 2019-01-05

**Authors:** Soon Hong Soh, Lai Yeng Lee

**Affiliations:** 1 Newcastle Research and Innovation Institute, 80 Jurong East Street 21, #05-04 Devan Nair Institute for Employment & Employability, Singapore 609607, Singapore; S.H.Soh1@newcastle.ac.uk; 2 Newcastle University in Singapore, 537 Clementi Road, #06-01 SIT Building@Ngee Ann Polytechnic, Singapore 599493, Singapore

**Keywords:** supercritical carbon dioxide, microencapsulation, microporous foam, supercritical drying, supercritical anti-solvent

## Abstract

The unique properties of supercritical fluids, in particular supercritical carbon dioxide (CO_2_), provide numerous opportunities for the development of processes for pharmaceutical applications. One of the potential applications for pharmaceuticals includes microencapsulation and nanoencapsulation for drug delivery purposes. Supercritical CO_2_ processes allow the design and control of particle size, as well as drug loading by utilizing the tunable properties of supercritical CO_2_ at different operating conditions (flow ratio, temperature, pressures, etc.). This review aims to provide a comprehensive overview of the processes and techniques using supercritical fluid processing based on the supercritical properties, the role of supercritical carbon dioxide during the process, and the mechanism of formulation production for each process discussed. The considerations for equipment configurations to achieve the various processes described and the mechanisms behind the representative processes such as RESS (rapid expansion of supercritical solutions), SAS (supercritical antisolvent), SFEE (supercritical fluid extraction of emulsions), PGSS (particles from gas-saturated solutions), drying, and polymer foaming will be explained via schematic representation. More recent developments such as fluidized bed coating using supercritical CO_2_ as the fluidizing and drying medium, the supercritical CO_2_ spray drying of aqueous solutions, as well as the production of microporous drug releasing devices via foaming, will be highlighted in this review. Development and strategies to control and optimize the particle morphology, drug loading, and yield from the major processes will also be discussed.

## 1. Introduction

Well-established processes using supercritical CO_2_ in pharmaceutical applications include micronization by RESS (rapid expansion of supercritical solutions), SAS (supercritical antisolvent), or ScMM (supercritical melt micronization), microencapsulation via co-precipitation (in RESS, SAS, supercritical spray drying, etc.), active ingredient coating (spray coating, supercritical CO_2_ fluid bed coating, etc.), sterilization (due to microbial inactivation properties of pressurized CO_2_), biopolymeric microporous foam/sponges (supercritical foaming, supercritical impregnation, etc.). Many review and research articles have been published on the topic of using supercritical fluid techniques for the development of drug delivery [1,2,3,4,5,6,7], biomedical and pharmaceutical formulations or devices [8,9]. Supercritical fluid can be used in many different ways to produce microencapsulated and nanoencapsulated products based on the properties of the active ingredient, coating material, and suitable solvent (if any) used.

The selection of processing technique with supercritical CO_2_ for biopolymers depend greatly on the interaction of the supercritical CO_2_ with the active ingredient, coating material of interest, and suitable solvent. In biopolymeric drug delivery systems, the interaction of the polymer with supercritical CO_2_ plays an important role in the selection of the supercritical process. For instance, polylactide (PLA) is a suitable candidate for SAS, but not RESS, as it is not easily soluble in supercritical CO_2_ [10,11]. On the other hand, polylactide-*co*-glycolide (PLGA) has a low glass transition temperature (*T*_g_), and the further depression of its *T*_g_ in the presence of supercritical CO_2_ makes it difficult to produce discrete free-flowing powders via the SAS process. However, this property of PLGA makes it suitable for the production of microporous polymeric foams with very low residual organic solvent content, allowing the production of microporous drug-releasing polymeric foams that can be used as implant and scaffold material [12,13,14].

Well-established methods for drug microencapsulation and nanoencapsulation for drug delivery include solvent evaporation, emulsion techniques, spray drying, and electrospray. Due to its attractive properties as a clean and green solvent for processing drugs and biopolymers, supercritical CO_2_ processing has been regarded as an important option for the production of microencapsulated drug delivery devices [15].

The role of supercritical CO_2_ (as a solvent, antisolvent, or solute) in particle design, pharmaceutical ingredient processing, and composite particle production has been evaluated by earlier reviews [6,9,16,17,18]. In a more recent review by Padrela et al. [19], the production of nanoparticle and nanocrystals via supercritical carbon dioxide was evaluated with a comprehensive description of the selection criteria of different methods based on the properties of the active ingredient. This review will focus on the applications where the active ingredient is encapsulated in microsized, nanosized, or microstructured formulations with applications for pharmaceutical or drug delivery. This paper aims to provide the reader with a comprehensive review of the currently available options to design and produce microencapsulated and nanoencapsulated formulations ranging from microparticles to nanoparticles, and including recent developments such as fluidized bed coating using supercritical CO_2_ and the production of drug-releasing three-dimensional microporous foam for pharmaceutical applications. The discussion will introduce readers to different supercritical fluid processing techniques that utilize the favorable and versatile properties displayed by supercritical CO_2_, with an understanding of the underlying mechanism behind each method, and the modifications applied to improve the particle size, morphology, and process efficiency.

## 2. Supercritical Carbon Dioxide Processing Systems

Depending on the active ingredient or drug compound of interest, the type of formulation required (e.g., selection of suitable coating material for desired release profile), and the morphology of the final encapsulated product, it is possible to modify the configuration of the supercritical fluid system to perform different processes, as shown in our earlier studies where experiments for SAS [10,11,20], SAS with enhanced mass transfer (SAS-EM) [10], supercritical foaming [12,13,14,21], and supercritical drying [22] were performed with a custom-built supercritical fluid system.

The main sections for a supercritical CO_2_ unit for drug delivery production include: (i) a high-pressure CO_2_ delivery system; (ii) a secondary high-pressure delivery system; (iii) a high-pressure vessel(s)/high-pressure chamber(s); (iv) a product separation/collection/purification system. The basic equipment required for supercritical CO_2_ processing include: (1) a compressed liquid CO_2_ cylinder (preferably with a dipstick); (2) high-pressure liquid pumps for the delivery of CO_2_ to supercritical pressure, and for co-solvents or solutions to be delivered and in contact with high-pressure CO_2_; (3) chillers for liquefying, or ensuring that the CO_2_ at the pump head is in a liquid state; (4) high-pressure chambers/vessels that need to be designed according to specifications of high-pressure vessels with fittings and tubings rated for high-pressure applications (from HIP, Swagelok etc.); (5) pressure controllers (either automated back-pressure regulators or back-pressure valves; (6) product separation units, such as flash vessels, cyclone separators for solid–fluid separation, solid filter units, a zeolite-packed bed for removing moisture from water-saturated supercritical CO_2_; (7) heaters/coolers for temperature control at various stages of the process, and (8) a recirculating pump (if the CO_2_ is recycled). A detailed summary of supercritical fluid particle formation in the pharmaceutical industry and representative vendor for supercritical fluid equipment and accessories can be found in the work of Vemavarapu et al. [23].

## 3. Role of Supercritical Carbon Dioxide in Microencapsulation and Nanoencapsulation

**Figure 1:**
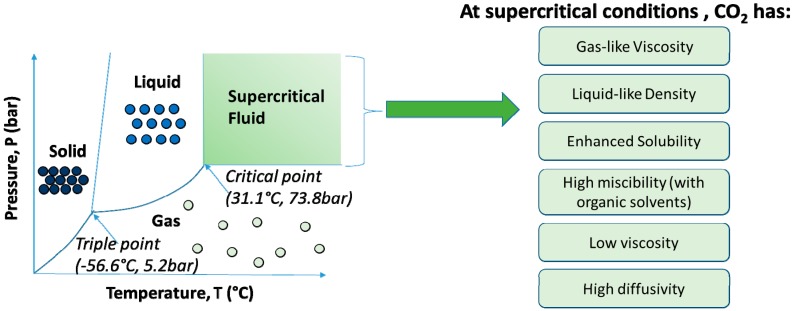
Phase diagram of carbon dioxide (not to scale) and its properties at supercritical conditions.

CO_2_ has relatively accessible critical conditions of 73.8 bar and 31.1 °C. Its low critical temperature allows processes to be developed at close to ambient temperatures (e.g., at 35 °C). Other favorable qualities of supercritical CO_2_ include its non-toxic and non-flammable nature, gas-like viscosity, liquid-like density with enhanced solubility, microbial inactivation abilities, and relatively low cost. Figure 1 summarizes the properties of supercritical CO_2_, which allow it to be applied for various microencapsulation and nanoencapsulation processes. The selection of the processing techniques used for microencapsulation and nanoencapsulation depends on the materials and thermodynamic properties of the active ingredient, the coating material, and any suitable co-solvent that is available. Other criteria in the selection of a suitable supercritical fluid technique include the desired formulation (size, morphology, release profiles, porosity, etc.).

The role of supercritical CO_2_ as a solvent, antisolvent, solute, drying medium, and foaming agent will be evaluated and explained in the following sections.

### 3.1. Supercritical Carbon Dioxide as a Solvent

At supercritical conditions, CO_2_ has enhanced solubility for substances such as essential oils, active ingredients in plants and natural products [24,25,26], small molecular weight non-polar compounds, and low-molecular weight biopolymers. As supercritical conditions can be reached even at low temperature, this makes supercritical CO_2_ very attractive as a solvent, and it has been applied in numerous studies for the extraction of active ingredients (e.g., for caffeine decaffeination, essential oil extraction, active ingredient encapsulation, etc.).

#### Rapid Expansion of Supercritical Solutions (RESS)

**Figure 2:**
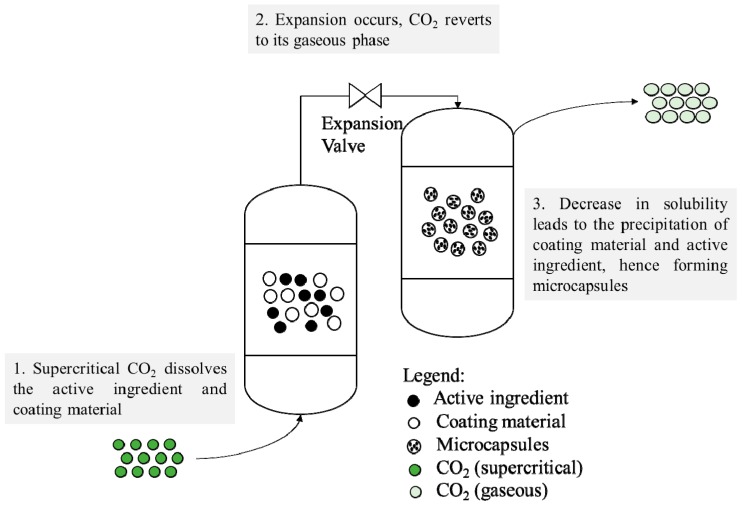
Schematic representation of the rapid expansion of supercritical solution (RESS) process.

In the rapid expansion of supercritical solutions (RESS), the active ingredient and coating ingredient are dissolved in a supercritical fluid (acting as a solvent). The supercritical fluid solution containing the solutes is maintained at high pressure before expanding across a fine throttling device, such as a capillary or orifice nozzle [27,28,29]. At this point, supersaturation occurs, leading to the desolvation of the coating material, which is then deposited around the active ingredient, forming microcapsules (Figure 2). In active pharmaceutical ingredient (API) encapsulation applications, the microcrystalline pharmaceutical dominates the core of the particles, while the slower precipitating polymer coats the surface. The advantages of RESS include its capacity for a wide range of inorganic, organic, and polymeric materials, low-temperature operation, and single-step processing [30]. Table 1 shows some examples of bioproducts that are encapsulated using the RESS process. The prerequisite of this process is that both the active ingredient and the coating material must be very soluble in supercritical fluids. This typically applies to lower molecular weight polymers and small active ingredients such as non-polar small molecule compounds.

The RESS of polymer solutions in CO_2_ has been limited by low polymer solubility at temperatures below 80 °C [31]. To overcome this limitation, the RESS process can be modified with the application of a co-solvent. The solubility of the polymers in CO_2_ increases significantly with low molecular weight alcohols as co-solvents, such as ethanol and methanol [32]. The modified process is termed as rapid expansion of supercritical solutions with a non-solvent (RESS-N). In the RESS-N process, a suspension of the active ingredient in a co-solvent containing CO_2_ and a dissolved polymer is sprayed through a nozzle to atmospheric pressure. The co-solvent, in pure form, is a non-solvent for the polymer, and is only sparingly soluble in the polymer particles that are produced during expansion [33]. Therefore, the particles do not agglomerate after expansion, since there is no swelling of polymer products. The modified RESS has been applied to the encapsulation of proteins [32] and medicines [34], as shown in Table 2. The polymer-coating thickness, mean particle diameter, and particle size distribution of microcapsules could be controlled by changing the feed composition of the polymer [32,34].

### 3.2. Supercritical Carbon Dioxide as an Anti-Solvent

The limitations of RESS can be overcome by a supercritical antisolvent (SAS), which utilizes the high miscibility of supercritical CO_2_ with organic solvents; this can be used to dissolve both the active ingredient and the coating material of interest. When the organic solution is introduced into supercritical CO_2_, the CO_2_ rapidly extracts the organic solvent from the solution jet, leading to the rapid precipitation of the composite product. The resulting product will be an active ingredient distributed in the matrix of the coating material.

#### 3.2.1. Supercritical Antisolvent (SAS)

The supercritical antisolvent (SAS) technique is one of the most versatile [35] and widely researched techniques using supercritical CO_2_ for micronization and microencapsulation [10,35,36,37,38,39,40,41,42,43,44,45,46,47,48,49,50,51,52]. The process can be applied to a wide range of compounds and biopolymers that have limited solubility in supercritical CO_2_. The SAS process has been applied in micronizing APIs, and has been shown to produce improve bioavailability and the solubility of hydrophobic drug compounds by size reduction and the control of crystal morphology [41]. Different biopolymeric morphologies (threads, sponges, and microparticles) can also be achieved from SAS processes [49]. Although the SAS is favorable for the production of formulations for hydrophobic drugs with low bioavailability and poor aqueous solubility [41,44,47,53], the encapsulation of hydrophilic compounds [40,54] have also been demonstrated by dispersing the hydrophilic drug in the organic solution. The co-precipitation produces monolithic matrix systems rather than reservoir systems, and the drug release profiles typically follow diffusion-controlled or polymer degradation mechanisms [10].

**Figure 3:**
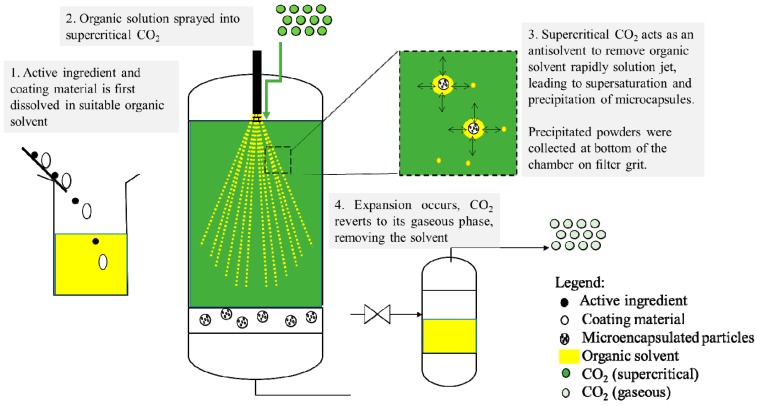
Schematic representation of supercritical antisolvent (SAS).

Typically, SAS operates by the continuous and simultaneous injection of the organic solution with supercritical CO_2_ into a chamber with supercritical CO_2_ via a nozzle. The mechanism of particle formation using SAS is illustrated in Figure 3. The means of contact between the solution and CO_2_ can vary in different versions of the process, leading to several variations in the SAS process such as the PCA (precipitation from compressed antisolvent) [55], ASES (aerosol solvent extraction system) [50], SEDS (solution-enhanced dispersion by supercritical fluids) [42], and SAA (supercritical-assisted atomization) [53]. In the SEDS process, the solution and supercritical CO_2_ were introduced into the precipitation vessel simultaneously via a co-axial nozzle. This enhanced the dispersion and contact between the solution and supercritical CO_2_. Chattopadhyay and Gupta [56] designed a method of combining ultrasonic mixing within a SAS chamber to achieve nanoparticle and nanoencapsulations [54] via an enhanced mixing mechanism between supercritical CO_2_ and the organic solution. These processes were designed and developed to achieve better understanding and control of the SAS process in order to obtain particles with desired characteristics [43,48,57]. In addition, numerous studies were also carried out to understand the underlying mechanism during SAS, such as the jet break-up phenomena [10,20,58,59], the role of mass transfer [46,56], the solvent–antisolvent interaction behavior, etc.

#### 3.2.2. Supercritical Fluidized Bed Coating

Fluidized bed drying and coating processes have been well established as scalable means of obtaining coated APIs (e.g., Wurster fluid bed coating) [60]. The use of supercritical CO_2_ as the fluidizing medium for core particles as well as the drying agent or antisolvent to remove the moisture or solvent respectively from core particles coated with the coating material solution is of great interest, as it allows the coating to be performed in oxygen-free and low-temperature conditions.

In the studies by Subramaniam et al. [61], a Wurster-type coater employing near-critical CO_2_ as an antisolvent for solvent removal from coated particles was developed. Studies on particle fluidization with supercritical CO_2_ have shown that conventional correlations for fluidization can be applied for supercritical fluids for the prediction of minimum fluidizing and terminal velocities [62]. Supercritical fluidized bed coating utilizes the fluidization of solid core particles using supercritical CO_2_ as a fluidizing medium and at the same time, a solution with the coating material will be sprayed onto the fluidized particles. The supercritical CO_2_ plays the role of a drying medium (for aqueous solutions) or an antisolvent (for organic solutions) to dry the fluidized particles in the bed. The coating of API and ingredients such as curcumin has been demonstrated using supercritical fluid coating processes [61,63,64,65]. This technique has promising development for the production of controlled multi-layered coatings on APIs to achieve formulations with desired sustained release or surface properties.

#### 3.2.3. Supercritical Fluid Extraction of Emulsions (SFEE)

**Figure 4:**
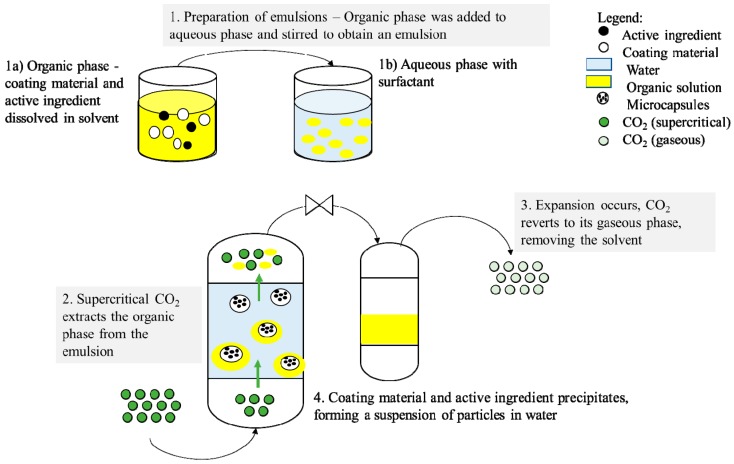
Schematic representation of supercritical fluid extraction of emulsions (SFEE).

One of the limitations of RESS and SAS in microencapsulation is the processing of polymers that tend to plasticize in the presence of supercritical CO_2_, including the class of commonly used amorphous polymers in drug delivery (including PLGA, polymethylmethacrylate (PMMA), and polycaprolactone (PCL)) [66]. To overcome the above-mentioned limitations, Chattopadhyay and Shekunov [66] presented the supercritical fluid extraction of emulsions (SFEE) technique. In this technique, the active ingredient and polymer is dissolved in organic solvent, and the organic phase and the organic solution are then emulsified with aqueous phase to form an oil/water emulsion. Supercritical CO_2_ is used to extract the organic solvent from the emulsion, leading to the supersaturation of the active ingredient and polymer in the aqueous phase, and resulting in the precipitation of the active ingredient and polymer. The precipitated microencapsulation or nanoencapsulation particles are subsequently collected from the aqueous phase. Figure 4 illustrates the general schematic representation of the SFEE process.

An important feature of the SFEE process is the ability to form nearly monodisperse microencapsulates or nanoencapsulates. Table 3 shows examples of microencapsulated and nanoencapsulated formulations produced using the SFEE process. Active ingredients ranging from low-bioavailability hydrophobic drugs [66,67,68] to model proteins [69] and even oils [70] can be encapsulated using this method.

### 3.3. Supercritical Carbon Dioxide as a Drying Agent

CO_2_ has an increased affinity for water at conditions above its critical point (31.1 °C, 73.8 bar) [73], which makes it a good candidate for drying aqueous solutions and wet samples that otherwise cannot be dried by traditional drying techniques due to their thermal sensitivity and oxidation. Supercritical CO_2_ has been found to be a good drying medium for food matrices [74,75,76,77], aerogels [78,79,80,81,82], and other natural products [22] due to its offer of low processing temperatures.

#### Supercritical Spray Drying 

**Figure 5:**
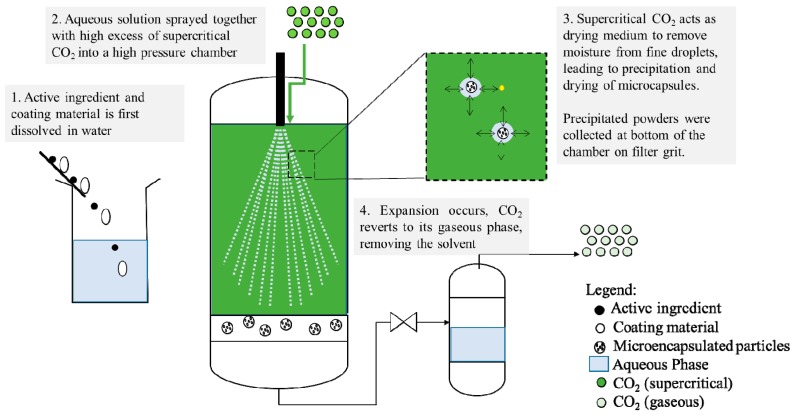
Schematic representation of supercritical spray drying.

Supercritical spray drying refers to the spraying of an aqueous solution into an excess of supercritical CO_2_. Similar to hot air spray-drying, supercritical CO_2_ spray drying utilizes the increased solubility of CO_2_ with water at supercritical conditions. The break-up of the aqueous solution into very tiny droplets (with high surface area to volume ratios) enhances the mass transfer of water into the supercritical CO_2_ in the drying chamber and therefore, the water is removed continually from the drying chamber. Figure 5 shows the schematic representation of the supercritical spray-drying process.

This technique is suitable in the microencapsulation formulations where the coating material is a water-soluble material (such as sugars, starches, maltodextrin, etc.), which will form the aqueous phase that will be sprayed into supercritical CO_2_. The active ingredient can be dissolved in the same aqueous phase (proteins or hydrophilic compounds) or distributed in the aqueous phase via emulsification (oil, fatty acids, or organic solution) or suspension (fine solid particles). One promising application of the process is the encapsulation of oils to produce free-flowing oil encapsulated powders [51,83,84,85], which can be applied in the nutraceutical market for oil (polyunsaturated fatty acids (PUFAs), docosahexaenoic acids (DHAs), eicosapentaenoic acid (EPAs) etc.). The production of a water-insoluble phospholipid-rich oil has been reported in the World Intellectual Property Organization (WO) patent application (WO 2010014011 A1) [86], where a two-step supercritical spray drying followed by a supercritical antisolvent procedure (for coating) was performed to obtain a microencapsulated oil with a non-soluble coating. Units ranging from four liters to 10 liters have been developed for the laboratory to demonstration scale of supercritical spray drying, where a co-axial nozzle was used to introduce and break up the aqueous solution into finely dispersed droplets using scCO_2_ as the dispersing agent and drying medium [87].

### 3.4. Supercritical Carbon Dioxide as a Solute

Due to its low viscosity and high diffusivity, supercritical CO_2_ can diffuse very efficiently into solutions, polymer melts, and also fatty acids. Using this property of supercritical CO_2_, strategies for producing microencapsulated particles from an aqueous solution can be achieved.

#### Particles from Gas-Saturated Solutions (PGSS)

**Figure 6:**
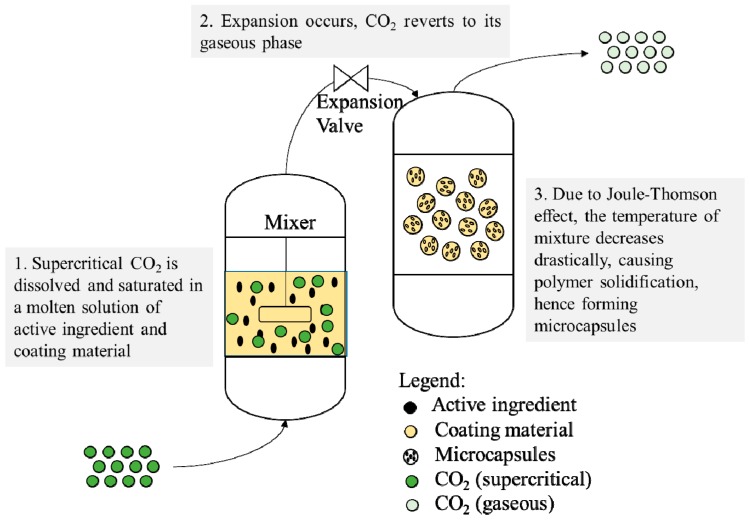
Schematic representation of particles from gas-saturated solutions (PGSS).

In particles from gas-saturated solutions (PGSS), supercritical CO_2_ acts as a solute, diffusing and dissolving into a melt or solution, forming a gas-saturated solution. The solution will then be expanded via a nozzle into a spray chamber at atmospheric pressure. The CO_2_ gas then leaves the gas-saturated polymer/fat droplets and also during expansion, the temperature of the mixture reduces drastically due to the Joule–Thomson effect, hence causing the polymer solidification [88]. A similar process termed as supercritical melt micronization (ScMM) has been developed for the micronization of fats (such as hard fats or milk fats) [89,90,91,92]. For microencapsulation application, the PGSS process can be used for water-soluble active ingredients and coating materials. The PGSS drying process (Figure 6) involves mixing an aqueous solution, containing the active material and wall material, and saturating the solution with supercritical CO_2_. Subsequently, the gas-saturated solution is expanded via a nozzle into a spray chamber at atmospheric pressure. This leads to the encapsulation of key compounds by the co-precipitation of the coating and core materials. PGSS holds several advantages over conventional methods such as coacervation, spray-drying, and emulsion techniques due to its mild operating conditions and its ability to produce solvent-free and homogenous products. This is especially beneficial in preserving the stability of ingredients such as essential oils [93,94] and heat-sensitive virus proteins [95], where elevated temperatures and organic solvents could cause negative interactions. Table 4 shows examples of bioproducts encapsulated via PGSS.

### 3.5. Supercritical CO_2_ as a Foaming Agent

In the production of microporous biopolymeric structures via supercritical CO_2_ foaming, supercritical CO_2_ is first contacted with the polymer in a high-pressure chamber. The supercritical CO_2_ diffuses into the polymer matrix, causing the glass transition temperature (*T*_g_) of the polymer to be lowered, and forming a solution of the polymer with CO_2_. On depressurization, the CO_2_ leaves the polymer–CO_2_ mixture and the polymer vitrify, leaving a microporous structure on the polymer. In this case, CO_2_ actually acts both as a solute and a foaming agent. This section will focus of the role of CO_2_ as a foaming agent to produce the microporous structure within the biopolymer matrix as a potential drug delivery platform for pharmaceutical applications.

The supercritical CO_2_ foaming of biopolymers is an attractive method for the production of microporous constructs for biomedical applications. Using supercritical carbon dioxide as a foaming agent, the use of organic solvents for the fabrication of the PLGA foams can be minimized or avoided, resulting in a zero to low-residual solvent product. The pore size and morphology of the PLGA foams can be controlled by factors such as the operating conditions of the foaming process (temperature, pressure), the rate of depressurization, and the nature of the selected co-polymer (polymer functional end groups, lactic to glycolic ratio, molecular weight, etc.). The encapsulation of active ingredients ranging from protein, anticancer drugs, chitosan, etc. has been demonstrated for potential applications for implant drug delivery for chemotherapy, scaffold material for cell cultivation, new carriers for DNA delivery, etc.

Strategies for encapsulating the active ingredient or drug in a microporous biopolymeric matrix include a single-step impregnation process, as presented by Cabezas et al. [102,103] and via a two-step encapsulation supercritical foaming process [12,13]. The supercritical foaming of biodegradable polymers such as polylactic-*co*-glycolic acid (PLGA) has potential applications for drug delivery and biomedical implants.

#### 3.5.1. Single-Step Impregnation and Foaming

In the single-step impregnation and foaming process to produce microporous biopolymeric foams with encapsulated ingredients, supercritical CO_2_ acts both as a solvent for the active ingredient and as a solute in infiltrating the polymer matrix. This is a promising technique where a residual solvent-free implant can be obtained at low processing temperatures, which can be particularly important for thermally-labile active ingredients [104]. Indomethacin [103] and 5-fluorouracil [102] have been encapsulated in microporous polymer foams using the impregnation and foaming processes.

One of the current limitations of this method is that the drug loading in the polymeric material is limited by the solubility of the solute in the supercritical CO_2_. To achieve higher drug loading and encapsulation efficiencies, and to be able to encapsulate a range of different active ingredients (hydrophobic, hydrophilic, etc.), a two-step process can be considered for the design of desired formulations.

#### 3.5.2. Two-Step Drug Encapsulation and Foaming 

**Figure 7:**
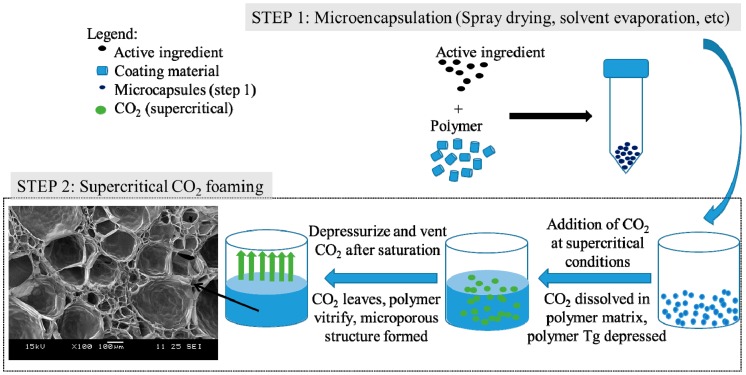
Schematic representation of two-step drug encapsulation and supercritical CO_2_ foaming.

The two-step process of drug encapsulation and foaming involves first obtaining a drug-encapsulated polymer matrix via methods such as solvent casting [105], spray drying [12,13], or emulsion methods [21]. The drug-encapsulated polymer then undergoes supercritical gas foaming to obtain a microporous polymeric structure with the drug encapsulated in the polymer matrix. The encapsulation of the active ingredient in the polymer matrix is not limited by its solubility in supercritical CO_2_, as the precursor drug-loaded polymer can be achieved by other well-established encapsulation methods such as solvent evaporation, emulsification methods, or spray drying (Figure 7).

In this method, most of the drug encapsulated in the polymer prior to foaming will remain in the microporous foamed product [12,21]. One drawback of the two-step encapsulation and foaming process is that an organic solvent is typically used in the first step. However, as CO_2_ is able to penetrate into the polymer matrix, the residual solvent is also removed by the CO_2_ during the foaming process, leaving behind a product with very low residual solvent content [12]. In our earlier studies, it was observed that the residual solvent in spray-dried particles of paclitaxel-loaded PLGA (using dichloromethane as the solvent) is reduced significantly after the CO_2_ foaming process [12]. Formulations encapsulating chitosan [13], paclitaxel [12], curcumin, and gentamicin [21] for applications as implants or scaffolds have been demonstrated using this method. The active ingredient will be encapsulated within the matrix of the microporous structure, and drug release will follow a diffusion mechanism and/or follow the degradation of the polymer matrix [12,106]. The microporous structure as a drug delivery device is particularly useful for the delivery of drugs with low bioavailability and low solubility. The high surface to volume ratio of the formulation enhances the drug release by diffusion [12,106].

## 4. Conclusions and Future Perspectives

The favorable and tunable properties of supercritical CO_2_ make it a very attractive option in processing products for pharmaceutical applications, particularly regarding the microencapsulation and nanoencapsulation of drugs or active ingredients for sustained or targeted release. The process that is selected depends on the properties of the active ingredient and coating material of interest, such as the solubility, hydrophobicity, molecular weight, glass transition temperatures, crystallinity, etc. The SAS method is very useful in the micronization and formation of amorphous drug particles. However, not all biopolymers can be processed using SAS due to the interaction of supercritical CO_2_ with the polymer. The SFEE technique provides an elegant alternative to the SAS process, which can be used to process a wider range of drug delivery polymers such as PCL, PLGA, PHBV, etc. Recent developments in combining a fluidization coating with SAS or supercritical drying also offer opportunities for the more specific design of a controlled-release formulation. The supercritical foaming technique allows three-dimensional microporous polymeric structures with encapsulated drugs to be produced. This provides opportunities to develop controlled release scaffold or implant materials.

The versatility and compatibility of supercritical fluid processing techniques also allow smart coating materials such as cyclodextrins to be used as encapsulating agents, which is useful in the nanoencapsulation and microencapsulation of flavors and aromas [107,108,109,110,111]. Formulations with cyclodextrins can also be produced for drug delivery applications, as demonstrated by Adeoye et al. for ibuprofen/hydroxypropyl-γ-cyclodextrin inclusion complexes via a supercritical CO_2_-assisted spray-drying process [112]. In our opinion, it will be important to develop integrated supercritical CO_2_ processing strategies that combine multiple steps in pharmaceutical processing. The highly tunable solvent properties of supercritical CO_2_ can be explored to develop processes for particle formation or encapsulation, the removal of impurities and residual solvents, and the separation and recovery of organic solvents in a single process train, which can help intensify the pharmaceutical production processes [16]. The offer of a “greener” route [16] using little to no organic solvents during pharmaceutical formulation processing, and a “cleaner” product whereby the efficient removal of the residual solvent by supercritical CO_2_, and its microbial inactivation abilities, makes supercritical fluid technologies very favorable for pharmaceutical manufacturing industries.

From the available literature, it can be seen that the mechanism and various configurations of supercritical CO_2_ processes has already been studied extensively, providing a well-established database for both thermodynamic (density, viscosity, solubility at different temperature, pressure conditions, etc.) [113,114,115,116] and fluid dynamic behavior (jet break-up, mass transfer, etc.) [58,59]. A focus on the research and development of systems in compliance with pharmaceutical manufacturing practices, with a clear evaluation of health and safety guidelines and considerations for operation, and complete with a techno-economic model of the technology, will help realize its potential for implementation and scale-up in pharmaceutical processes in the near future.

## Figures and Tables

**Table 1:** Examples of bioproducts encapsulated using the RESS process.

Active Ingredient	Coating Material	Particle Size and Morphology	Reference
Coenzyme Q_10_	Poly(ethylene glycol) (PEG) Polylactic acid (PLA)	2–10 µm PEG: Uniform and small spheres PLA: Spherical agglomerated microparticles	[27]
Felodipine	PEG4000	2–6 µm Gel-like irregular mass	[28]
Melatonin	Liposomes	66 nm Round or oval microspheres with uniform distribution	[29]
Naproxen	PLA	10–90 µm Microspheres with agglomerates	[30]

**Table 2:** Examples of microencapsulation applications by the rapid expansion of supercritical solutions with a non-solvent (RESS-N).

Active Ingredient	Coating Material	Co-Solvent	Reference
Proteins (lysozyme and lipase)	PEG4000, PEG6000, PEG20000, poly(methyl methacrylate) (PMMA), PLA, polyglycolide-*co*-lactide (PGLA), and PEG- poly(propylene glycol) (PPG)-PEG triblock copolymer	Ethanol/methanol/propanol/acetone/toluene	[32]
P-acetamidophenol, acetylsalicylic acid, 1,3-dimethylxanthine, flavone, and 3-hydroxyflavone	PEG4000, PEG6000, PEG20000, PLA, PMMA, ethyl cellulose, and PEG-PPG-PEG triblock copolymer	Ethanol/methanol/propanol/acetone/toluene	[34]

**Table 3:** Examples of microencapsulates and nanoencapsulates by the SFEE process.

Active Ingredient	Coating Material	Particle Size and Morphology	Reference
Indomethacin	Polylactide-*co*-glycolide (PLGA)/Eudragit RS	<1 µm Spherical	[66]
Lysozyme	PLGA	~0.1–1 µm Spherical	[69]
Ketoprofen	PLGA	~0.1–1 µm Spherical	[66,68]
Vitamin E	Polycaprolactone (PCL)	~10–300 nm Spherical nanoparticles	[71,72]
Medroxyprogesterone	Poly(3-hydroxybutirate-*co*-3-hydroxyvalerate) (PHBV)	~0.1–1 µm Spherical	[67]
Omega-3-rich fish oil	PCL	~10–10 nm Spherical nanoparticles	[70]

**Table 4:** Examples of bioproducts encapsulated using the PGSS process.

Active Ingredient	Coating Material	Particle Size and Morphology	Reference
β-carotene	Soy lecithin	10–500 μm Agglomerates of partially fused spheres	[96]
	Polycaprolactone (PCL) (CAPA 2403D and CAPA 6100)	CAPA 2403D: 110–130 μm CAPA 6100: 270–650 μm Flat or sphere-like particles attached and agglomerated by long filaments of polymer	[97]
Coffee oil	Polyethylene Glycol (PEG)	78 μm Spherical shapes of various sizes to amorphous shapes	[98]
*Cydia pomonella* granulovirus	Palm oil-based fat: 77% Lecithin-based surfactant: 9% Modified titanium oxide and benzophenone derivative UV protectants: 2%	<85 μm Almost spherical particles were obtained	[95]
Lavandin essential oil	PEG 9000	30–100 μm Spheres and needles	[93]
	Soy lecithin	1.4–24.8 μm Dry and fine but aggregated particles	[94]
Limonene	Modified starch	60–90 μm Spherical shapes with few broken shapes smaller than the others	[99]
Omega-3 polyunsaturated fatty acids and astaxanthin-rich salmon oil	PEG 6000	67.26–165.81 μm Irregular spherical shapes to amorphous shapes of various sizes	[100]
Quercetin	Soy lecithin and Pluronic L64^®^	0.138–0.158 μm Complete encapsulation of amorphous quercetin	[101]
